# Sleep, Anxiety, and Academic Performance: A Study of Adolescents From Public High Schools in China

**DOI:** 10.3389/fpsyg.2021.678839

**Published:** 2021-07-01

**Authors:** Xiaoning Zhang, Dagmara Dimitriou, Elizabeth J. Halstead

**Affiliations:** Department of Psychology and Human Development, University College London, Institute of Education, London, United Kingdom

**Keywords:** sleep, China, anxiety, pandemic, learning, mental health

## Abstract

**Purpose:** Sleep is essential for optimal learning across the developmental pathways. This study aimed to (1) explore whether school start and end times and screen time influenced sleep disturbances in adolescents during the lockdown in China and (2) investigate if sleep disturbances at night and sleep-related impairment (daytime fatigue) influenced adolescents' academic performance and anxiety levels.

**Methods:** Ninety-nine adolescents aged 15–17 years old were recruited from two public schools in Baishan City Jilin Province, China. An online questionnaire was distributed including questions on adolescents' demographics, screen time habits, academic performance, anxiety level, sleep disturbances, and sleep-related impairment.

**Results:** Adolescents who started school earlier and ended school later had a greater severity of sleep disturbances and sleep-related impairment compared with students who started school later and finished earlier. Adolescents who engaged in screen time at bedtime were more likely to have a greater severity of sleep-related impairment than students who reported no screen time use at bedtime. Adolescents who had a greater severity of sleep disturbances had higher anxiety and higher academic achievements than adolescents with less sleep disturbances. Finally, 79.7% of adolescents reported their total sleep duration as <8 h.

**Conclusions:** Adolescents are experiencing sleep disturbances to manage academic demands during COVID-19, which in turn is having a wider impact on their mental health. Many schools internationally have continued to provide online education to students, longitudinal studies on how COVID-19 has influenced adolescents sleep and mental health would be beneficial in understanding the impact of the pandemic.

## Introduction

### Environmental Factors and Adolescents' Sleep: School Start and End Times, and Screen Time

Sleep in adolescence has been characterized by social, environmental, and cultural factors (Owens, 2004). Environmental factors, such as school start/end times and the increased use of mobile devices, have been previously identified as affecting adolescents' sleep duration leading to greater daytime fatigue (Borlase et al., 2013; Hale and Guan, 2015). Research in the US has demonstrated that a 30-min school start time delay is associated with improved nocturnal sleep duration and reduced napping in adolescents (Knutson and Lauderdale, 2009; Owens et al., 2010). In addition, adolescents from schools with a later school start time were less likely to feel tired during classroom lessons and had increased daytime alertness than adolescents from schools with an earlier start time (Gariépy et al., 2017; Lo et al., 2018).

Several studies have found that increased use of mobile devices has resulted in a shorter sleep duration, longer sleep onset latency, increased sleep deprivation, later bedtime, and a delayed wake up time (Punamäki et al., 2007; Hysing et al., 2015). Screen time at bedtime could affect adolescents' circadian rhythm by suppressing the production of sleep-promotion melatonin (Higuchi et al., 2005; Gooley et al., 2011).

### The Role of Sleep Quality on Adolescents' Academic Performance and Emotion

Associations between adolescents' sleep quality and their academic performance and mental health have been previously identified. Adolescents who have a shorter sleep duration and greater sleep disturbances have been found to have lower academic achievements than those with longer sleep hours and less sleep disturbances (Gibson et al., 2006; Mak et al., 2012; Titova et al., 2015). In addition, individuals with sleep disturbances have been found to be at higher risk of having trait anxiety than those without sleep disturbances (Breslau et al., 1996; Neckelmann et al., 2007). The education system in China aims to produce high- achieving students, and has been widely criticized by researchers and educators for producing excessive pressure leading adolescents to sacrifice their sleep time to achieve high academic performance (Yang et al., 2007; Li et al., 2010; Zhao et al., 2014). In China, adolescents have been found to have high levels of both sleep deprivation and daytime sleepiness (Liu et al., 2008). In addition, high academic expectations encouraged by the educational system and parents has been found to be associated with greater anxiety and depression in adolescents in China (Quach et al., 2015).

### The Current Study

Due to COVID-19, ~170 countries enforced the closure of schools by the end of April 2020 (UNESCO, 2020) and many schools have continued to deliver online courses during the pandemic. In China, full lockdown began on the 23rd January and ended on the 8th April and schools provided online learning while schools were closed from February to September. The change to online learning led to an increased use of screen time for students and adjustments to school day structure and timings, yet few studies have focused on the impact of these changes on adolescents' well-being in China during the pandemic (Liu et al., 2005). One recent study explored how national lockdown has influenced 6- to 17- year-old children's screen time use during quarantine in Shanghai China. It was found that children and adolescents' total screen time had increased from 610 to 2,340 min per week, and their leisure screen time had increased by ~300 min per week (Xiang et al., 2020).

Typically, schools in rural China have a school day between 12 and 14 h, consisting of morning learning, daytime classes, and evening learning. The start time of schools did not change during the pandemic. Prior to February 2020, it was mandatory for students to remain in school until the end of the evening learning sessions, however, student's attendance for the evening sessions was not monitored during the pandemic, and therefore became optional for students to attend. This change from mandatory evening learning in school, along with increased use of technology to facilitate online learning during COVID-19, is likely to have affected adolescent sleep and mental health. Both sleep and mental health are important for maintaining resilience and continued learning (Majumdar et al., 2020).

This study aimed to (1) explore whether school start and end times and screen time influenced sleep disturbances in adolescents during the lockdown in China and (2) investigate if sleep disturbances at night and sleep-related impairment (daytime fatigue) influenced adolescents' academic performance and anxiety levels. In this study, sleep disturbance encompassed difficulties with falling and remaining asleep, as well as perceptions of sleep adequacy and satisfaction (PROMIS, 2021). Sleep-related impairment focused on self-reported alertness, sleepiness, and tiredness during normal waking hours, as well as perceived functional impairments during wakefulness associated with sleep disorders or diminished alertness (PROMIS, 2020).

## Methodology

### Participants

The online survey was distributed to all Year 11 students (~400) via two public schools who consented to participating in the research, located in Baishan City Jilin Province, China. Participation in the survey was voluntary. One year group was selected to obtain consistency between academic performance scores. Initially, 113 students consented to participating in the study, of these 99 participants aged between 15 and 17 years old (*M* = 15.83, *SD* = 0.67) fully completed the online survey and were included as the final sample. Students across schools in same city are ranked academically using their final examinations, therefore all participants were asked to provide their average academic ranking within their year group for comparison. Participants highest academic ranking ranged from 2nd place to 400th place (*M* = 102.54, *Mdn* = 73.00, *SD* = 89.33). A lower academic ranking value reflected a higher score in the final examination.

### Procedure

Prior to conducting this study, ethical approval was gained from [removed for blind peer review] Research Ethics Committee and the survey was piloted on five high school students in China, who were matched on educational background to the proposed participants to ensure survey length and presentation were acceptable. All the materials were translated and back translated (Brislin, 1970) into Mandarin Chinese. Once informed consent from parents was obtained, the head teacher of each school sent a secure electronic letter including the study information sheet, informed consent, and a link to the survey via Qualtrics^XM^ (https://www.qualtrics.com) to each student. Due to this study being completed during the 2020 pandemic and schools were closed, students were required to use electronic devices to participate in online learning. Each head teacher distributed the Qualtrics survey link to potential participants (see Participants) via a secure chat group within WeChat. WeChat is the most widely used social media in China which has more than 1 billion users monthly (CNNIC, 2016), it is also the preferred method for student-teacher correspondence electronically. Participants were asked to complete the consent form and survey voluntarily.

### Instrument and Measures

The online questionnaire included three validated measures in Mandarin Chinese in addition to a back translated author created demographic and screen time use questionnaire.

#### Background Information Questionnaires

Demographic information included; diagnosis (physical and mental health conditions, and learning difficulties), school start and end time, and academic ranking within the city.

#### Screen Time Use Questionnaires

This questionnaire included questions on adolescents' screen time habits in the last 7 days including frequency, timing, choice of electronic devices, and purpose of the screen time. The questions were answered as multiple choice. Example questions included “What devices do you have used in the past 7 days?”; “What time of day do you usually engage in screen time?”; “What do you use screen time for?”.

#### Anxiety

Participants' anxiety in general has measured using the State-Trait Anxiety Inventory (STAI-T) (Spielberger et al., 1983). The STAI-T is a self-report measure to evaluate participants' symptoms of anxiety experienced day to day. Participants are asked to respond to statements such as “I feel pleasant” and “I lack self-confidence.” The STAI-T measure has previously been translated into Mandarin Chinese has been found to have good reliability and validity in use with adolescents (Li and Lopez, 2004). Responses on the 20-item scale are measured by a 4 point Likert scale (1 “*Almost Never”* to 4 “*Almost Always”*). Total STAI-T scores range from 20 to 80 points and some items are reversed scored. A higher total score of items indicated higher anxiety. In this study, the Cronbach's alpha of STAI-T was 0.90 which indicated high internal consistency.

#### Sleep Disturbance

The PROMIS Pediatric Sleep Disturbance short form 8a (Buysse et al., 2010) was used to measure children's sleep depth, quality, and restoration in the last 7 days. Example questions included “In past 7 days, I had difficulty falling asleep.”; “In past 7 days, I tossed and turned at night.” Previous studies including adolescents in China have indicated good reliability (Ding et al., 2020). The measure includes 8-items with responses on a Likert scale ranging from 1 “*Never*” to 5 “*Always*.” Certain items were reversed scored and the total raw score of the 8-items was converted to a standardized T-score based on recommended scoring in the PROMIS scoring Manual (PROMIS, 2021). Participants' total T-score for sleep disturbances ranged from 38.54 points to 77.66 points. Higher scores demonstrated greater severity of sleep disturbances. In this study, the Cronbach's alpha value of the raw scores was 0.91 which indicated high internal consistency.

#### Sleep-Related Impairment

The PROMIS Pediatric sleep-related impairment scale (Buysse et al., 2010) is a 13-item 5 points Likert scale (1-Never; 5-Always) that investigates children's sleepiness, tiredness, alertness, and functional impairment related to sleep problems during waking hours in the last week. Participants responded to statements, for example “In past 7 days, I was sleepy during the daytime.”; “In past 7 days, I was in a bad mood because I was sleepy.” Participants' total raw score in sleep-related impairment was converted into standardized T-score for each participant based on recommended scoring in the PROMIS scoring Manual (PROMIS, 2020). The total T-score for sleep-related impairment ranged from 33.41 points to 76.99 points. The higher the T-score, the greater the sleep-related impairment. In this study, the Cronbach's alpha value of the raw scores was 0.96 which indicated high internal consistency.

### Statistical Analysis

IBM SPSS Statistics 26.0 were used to conduct all statistical analysis. Tests of normality were undertaken to explore the distribution of data. There were six variables in this study including (1) school start/end time, (2) screen time duration and timing, (3) sleep disturbance, (4) sleep-related impairment, (5) academic performance/ranking and (6) anxiety level. Shapiro-Wilk test indicated that the academic ranking (*W (99)* = 0.88, *p* < 0.05), sleep disturbances scores (*W (99)* = 0.92, *p* < 0.05), and sleep-related impairment scores (*W (99)* = 0.97, *p* < 0.05) outcome measures were not normally distributed. A Shapiro-Wilk test demonstrated that participants' anxiety scores were normally distributed (*W (97)* = 0.98, *p* = 0.361). Therefore, both parametric and non-parametric tests were conducted depending on the outcome measure included. Mann-Whitney tests were conducted to test for differences between school start times, screen time, and sleep disturbances and impairment scores. Spearman's Rho correlations and simple linear regressions were conducted to explore the associations between screen time, screen use, academic performance, anxiety, and sleep disturbances and sleep-related impairment. An a priori G^*^Power analysis (Faul et al., 2007) yielded that at least 27 participants were needed to detect a medium effect size (*f* ^2^ = 0.49) for linear regression design with a power of 0.80 α = 0.05).

## Results

Of the sample, 30.3% of participants were males and 69.7% were females. Overall, 49 participants were recruited from School 1 (49.5%) and 50 participants were recruited from School 2 (50.55%). The National Sleep Foundation (Hirshkowitz et al., 2015) released sleep duration recommendations for adolescents: (1) Not Recommended – <7 h; (2) Appropriate – 7–8 h; (3) Recommended – 8–10 h. Based on this criterion, participants in this study were categorized into three groups: (1) Short Sleep Duration Group: 2~4 h and 4~6 h; (2) Average Sleep Duration Group: 6~8 h; (3) Long Sleep Duration Group: over 8 h, 79.7% of adolescent's sleep duration was <8 h. Participants' average nocturnal sleep duration is reported in Table 1.

**Table 1:** Participants nocturnal sleep duration.

		**Frequency (*N*)**	**Percentage**
Sleep Duration (every night)	2~4 h	1	1%
	4~6 h	35	35.4%
	6~8 h	43	43.4%
	Over 8 h	20	20.3%

### School Start/End Time and Sleep

Two schools were included in the study and had different start and end time to each other. School [A] started 20 min earlier, and finished 70 min later, than school [B]. Sleep disturbances (*Mdn* = 52.51, *SD* = 10.80) and sleep-related impairment (*Mdn* = 51.85, *SD* = 10.25) were higher in School [A] than School [B] (*Mdn* = 45.53, *SD* = 7.92; *Mdn* = 45.14, *SD* = 7.96; *U* = 778, *z* = −3.14, *p* < 0.05, *r* = 0.32; *U* = 664, *z* = −3.93, *p* < 0.001, *r* = 0.39) indicating the longer school day impacted both student sleep disturbances and sleep-related impairment.

### Screen Use, Timing, and Sleep

Participants reported their overall daily screen time usage between 0~2 h (20.2%), 2~4 h (25.3%), 4~6 h (16.2%), and over 6 h (38.4%). Simple linear regressions were undertaken to predict participants' sleep disturbances and sleep-related impairment scores based on screen time. Scores derived from sleep scales demonstrated that the amount of time spent using electronic devices every day did not explain a significant amount of the variance in sleep disturbances *F*_(1, 97)_ = 0.18, *p* = 0.672, *R*^2^ = 0.002, *B* = −0.37) and sleep impairment score (*F*_(1, 97)_ = 0.088, *p* = 0.767, *R*^2^ = 0.001, *B* = −0.26).

Participants reported screen exposure via the use of electronic devices in the morning (42.4%), at lunchtime (45.5%), in the afternoon (50.5%), evening (67.7%), and at bedtime (51.5%). A Mann-Whitney test indicated that sleep impairment scores were greater for participants who reported screen use at bedtime (*Mdn* = 51.01, *SD* = 10.97) than for participants who reported no screen use at bedtime (*Mdn* = 47.66, *SD* = 8.41), *U* = 938, *z* = −2.00, *p* < 0.05, *r* = 0.20.

### Sleep and Academic Performance

Lower academic ranking values were significantly associated with higher sleep-related impairment (*r*_*s*_ (97) = −0.21, *p* < 0.05) and longer sleep duration (>8 h) (*r*_*s*_ (18) = −0.52, *p* < 0.05). A lower academic ranking reflected a higher academic performance. For example, if there are 400 students in Year 11 in total, and a student achieved the highest score for their final examination, their ranking would be “1.” Similarly, the lowest score in the cohort would be “400.” Students with high sleep-related impairment scores and longer sleep duration had better academic performance than those with low sleep-related impairment scores or shorter sleep duration. A simple linear regression was undertaken to predict participants' academic ranking based on their sleep-related impairment scores (*F*_(1, 97)_ = 6.36, *p* < 0.05, *R*^2^ = 0.062). As sleep-related impairment scores increased (by an increment of 1), participants' predicted academic ranking value decreased by 0.028. There was no significant association between academic ranking and sleep disturbance scores (*r*_*s*_ (97) = −0.06, *p* = 0.556), (*F*_(1, 97)_ = 1.67, *p* = 0.200, *R*^2^ = 0.017).

### Sleep and Anxiety

Increased anxiety was significantly associated with both increased sleep disturbances (*r*_*s*_ (95) = 0.52, *p* < 0.001) and sleep related impairment (*r* = 0.59, *p* < 0.001). Simple linear regressions found participants' sleep disturbances scores (*F*_(1, 95)_ = 41.21, *p* < 0.001) and sleep related impairment scores (*F*_(1, 95)_ = 53.67, *p* < 0.001) both significantly predicted their anxiety scores. Sleep disturbance explained a total of 30.3% of the variance in the model and sleep impairment explained a total of 36.1% of variance in the model. As anxiety scores increased (by an increment of 1), participants' predicted sleep disturbance scores increased by 0.53. Similarly, as participants' predicted anxiety score increased by 0.58, sleep-related impairment scores increased (by an increment of 1). No significant correlation was found between anxiety and academic ranking (*r* = −0.02, *p* = 0.84). See Tables 2, 3.

**Table 2:** Regression analysis for sleep disturbance and anxiety.

**Variable**	**B**	**95% CI**	**β**	***t***	***p***
(Constant)	17.71	[8.64, 26.79]		3.87	0.000
Sleep disturbance score	0.57	[0.40, 0.75]	0.55	6.42	0.000

*R^2^ adjusted = 0.30. CI = confidence interval for B*.

**Table 3:** Regression analysis for sleep-related impairment and anxiety.

**Variable**	**B**	**95% CI**	**β**	***t***	***p***
(Constant)	15.22	[6.58, 23.87]		3.50	0.001
Sleep-related impairment score	0.63	[0.46, 0.80]	0.60	7.33	0.000

*R^2^ adjusted = 0.35. CI = confidence interval for B*.

## Discussion

The current study found that most participants did not meet the national recommended sleep duration for adolescents (79.3%), which is an average of 8–10 h (Hirshkowitz et al., 2015). Participants who started school earlier and ended school later had a greater severity of sleep disturbances and sleep-related impairment compared with students who started school later and finished earlier. This finding supports previous research suggesting that a delayed school start time is beneficial for adolescents sleep quality and daytime functioning (Owens et al., 2010; Borlase et al., 2013). In China, particularly secondary cities and rural areas, school days are long, averaging over 12 h (Wei, 2014). Most previous research focuses on large cities such as Beijing and Shanghai, however, this study provided evidence of the impact of long school days in the secondary cities of China.

This study found that participants who engaged in screen time at bedtime were more likely to have a greater severity of sleep-related impairment than students who did not engage in screen time at bedtime. This finding supports previous research which indicated that screen time before sleep may lead to poorer sleep quality (Punamäki et al., 2007; Hysing et al., 2015). There were no association between participants' overall screen time duration each day and the severity of sleep disturbances and sleep-related impairment. This finding suggests that the timing of the use of screens at bedtime it is contributing factor in increased sleep disturbances and sleep-related impairment.

The current study indicated that students with a greater severity of sleep-related impairment had better academic performance than students with lower sleep-related impairment score. However, no association was found between sleep disturbances and academic performance. These results are contradictory to previous suggestions that adolescents with greater sleep disturbances were highly likely to have lower scores in their exams (Mak et al., 2012; Titova et al., 2015). However, it is important to consider this finding within the cultural context of China. Adolescents in China are taught to concentrate on obtaining high academic results. The cultural influence of Confucianism and Gaokao has encouraged students in China to sacrifice sleep to achieve high scores in exams. Nonetheless, this study found that adolescents in China who had a greater severity of sleep disturbances and sleep-related impairment had higher anxiety than those with less sleep disturbances and sleep-related impairment. These findings are consistent with previous research, which suggests that sleep-related problems are related to higher trait anxiety (Alfano et al., 2009; Fuligni et al., 2019). This suggests that although students in China may achieve better academic performance at the expense of sleep, sleep disturbances and sleep-related impairment were found to predict anxiety in students, suggesting potential negative mental health consequences to this practice. Therefore, future research is encouraged to explore these three factors in the cultural context of China.

Although adolescents' academic scores were high, there has been considerable evidence of how sleep deprivation and impairment impacts cognitive functioning and learning (Havekes et al., 2012; Kreutzmann et al., 2015). In addition, cross cultural comparisons may highlight the academic culture in China and the impact this has on sleep and mental health outcomes. Further studies could include measures of cognitive functioning to further evaluate the relationship between sleep and academic performance.

## Limitations

There were some limitations that existed in the current study. Generalizability of the results should be considered with the context of countries with a similar education system to China. Participants were students from Jilin Province, which has a population of 27.46 million, only 2% of the current population in China (Worldmeter, 2020). There is a significant difference in educational systems between big cities and rural areas in China. For example, big cities have higher college admission rates and more highly qualified teachers than secondary cities and rural areas (Yang, 2020). However, it is important to consider the impact on rural schools in China. Overall, recruitment of a larger sample size would have strengthened this analysis and provided greater generalizability to the findings.

Moreover, the current study provides cross sectional data in the context of a pandemic, this has its limitations for drawing conclusions from the data. Without pre COVID-19 baseline data or follow up data, it is undetermined if these relationships were pre-existing or directly related to the environmental changes during the pandemic (e.g., increased screen time and academic pressure). Specifically, it is possible that screen use at bedtime is a lifestyle factor to be considered in reducing sleep-related impairment overall, however, bedtime screen use may have increased due to demands during COVID-19 related to longer school days of which students are expected to continue their academic work into the night. Similarly, this study demonstrates associations between academic performance, sleep impairment, and anxiety, however it should be noted that it cannot be determined if this is related to the COVID-19 pandemic or if these relationships were pre-existing.

## Conclusions

To conclude, this study demonstrated whether school start/end time and screen time could predict adolescents' sleep disturbances and sleep-related impairment in China. This study also investigated whether adolescents' sleep disturbances and sleep-related impairment were associated with academic performance and anxiety level. Under the influence of a cultural context and COVID-19, this study demonstrated a series of interesting findings among adolescents in China. To address sleep disturbances in adolescents in China, Cognitive Behavioral Therapy for insomnia may be beneficial, however, the cultural attitude toward sleep and the seemingly academic benefits of lack of sleep should be considered in any treatment of insomnia (Roberts et al., 2006). These findings might encourage further consideration for the availability and effectiveness of interventions to address sleep disturbances, and future research in adaptations to address cultural differences in China and in countries with similar educational systems internationally. Further research should consider determining if these findings exist during the COVID-19 pandemic or continue post pandemic to support adolescent well-being in China.

## Implications and Contributions

This study investigated how school end time and screen time negatively influences adolescents' sleep. In addition, this study highlights how the timing of screen use in the evening impacts negatively on sleep quality. The cultural considerations of the pressures of academic performance and associated increased anxiety and sleep disturbances in adolescents in China, is discussed.

## Data Availability Statement

The raw data supporting the conclusions of this article will be made available by the authors, without undue reservation.

## Ethics Statement

The studies involving human participants were reviewed and approved by University College London, Institute of Education. Written informed consent to participate in this study was provided by the participants' legal guardian/next of kin.

## Author Contributions

XZ and EH conceived the study. XZ collected and analyzed the data. All authors edited and finalized the manuscript.

## Conflict of Interest

The authors declare that the research was conducted in the absence of any commercial or financial relationships that could be construed as a potential conflict of interest.
